# In Vivo Contactless, Cellular-Resolution Imaging of the Healthy and Pathological Human Limbus With 250-kHz Point-Scanning SD-OCT

**DOI:** 10.1167/tvst.13.12.29

**Published:** 2024-12-17

**Authors:** Kostadinka Bizheva, Zohreh Hosseinaee, Kirsten Carter, Denise Hileeto, Brian G. Ballios, Luigina Sorbara, Hall F. Chew

**Affiliations:** 1 Department of Physics and Astronomy, University of Waterloo, Waterloo, ON, Canada; 2 Department of System Design Engineering, University of Waterloo, Waterloo, ON, Canada; 3 School of Optometry and Vision Science, University of Waterloo, Waterloo, ON, Canada; 4 Department of Ophthalmology & Vision Sciences, University of Toronto, Toronto, ON, Canada; 5 Department of Ophthalmology & Visual Sciences, University of British Columbia, Vancouver, BC, Canada

**Keywords:** optical coherence tomography, ophthalmic OCT, limbus, palisades of Vogt, limbal crypts, limbal stem cell dysfunction, limbal stem cell dystrophy

## Abstract

**Purpose:**

To demonstrate that high-seed, ultra–high-resolution spectral-domain optical coherence tomography (SD-OCT) technology can image in vivo fine morphological features in the healthy and pathological human limbus.

**Methods:**

A compact, fiberoptic SD-OCT system was developed for imaging the human limbus. It combines ∼1.5-µm isotropic spatial resolution in ocular tissue and an acquisition rate of 250,000 A-scans per second. The imaging probe was outfitted with two microscope objectives to provide flexibility in the choice of wide field of view and extended depth of focus versus high lateral resolution. The clinical potential of the system was evaluated by imaging subjects with limbal stem cell dysfunction (LSCD; *n* = 4) and healthy controls (*n* = 6).

**Results:**

Limbus images acquired from the healthy controls showed normal cellular structure of the limbal crypts, palisades of Vogt (POVs), and vasculature of the underlying scleral tissue. Images acquired from the LSCD subjects showed distortions or absence of POVs, invasion of highly scattering conjunctival tissue over the limbal and peripheral corneal epithelium, scarring and thinning of the limbal epithelium, and neovascularization.

**Conclusions:**

The combination of high OCT spatial resolution and rapid image acquisition rate allows for in vivo, contactless, volumetric visualization of fine morphological details that could be beneficial for the precise diagnosis and grading of LSCD, planning of treatment, and evaluation of the effectiveness of the treatment approaches.

**Translational Relevance:**

The OCT technology described here could improve the clinical diagnostics and grading of LSCD, preoperative planning, and postoperative evaluation of LSCD subjects, in addition to monitoring the effectiveness of various LSCD treatments.

## Introduction

The human corneoscleral limbus is a tissue region about 1.5 mm wide that separates the peripheral cornea from the bulbar conjunctiva and sclera. The morphology of the limbus is very complex, as it contains ridges of fibrous tissue that extend either radially from the conjunctiva toward the peripheral cornea (palisades of Vogt [POVs]) or upward from the stroma toward the surface of the limbal epithelium (focal stromal projections), as well as blood and lymph vasculature and dense innervation.^1^^–^^7^ The limbus plays an important role in the regeneration of the corneal epithelium, as it houses the limbal stem cells (LSCs),^8^^–^^10^ which are located in limbal crypts^11^ bound by the POVs. The limbus also serves as a barrier between the corneal epithelium and the conjunctiva and prevents overgrowth of highly scattering conjunctival tissue into the corneal epithelium,^8^ as seen with pterygium development. The scleral part of the limbus contains both a rich blood vascular network, which is responsible for supplying oxygen and nutrition to the peripheral cornea, and a complex aqueous outflow system, which is responsible for waste management.^3^^,^^6^^,^^7^

Limbal stem cell dysfunction (LSCD) is a condition where the LSCs do not function properly and fail to generate new progenitor cells. LSCD results in decreased vision, photophobia, tearing, chronic inflammation, recurrent episodes of pain, progressive corneal conjunctivalization, and blindness in severe cases.^10^ LSCD can be caused by congenital factors such as aniridia,^12^ ectrodactylyl–ectodermal–dysplasia–clefting syndrome,^13^ keratitis–ichthyosis–deafness syndrome,^14^ xeroderma pigmentosum,^15^ dominant inherited keratitis,^16^ Turner syndrome,^17^ and dyskeratosis congenita.^18^ LSCD can also be related to infections such as herpes keratitis and trachoma. It can be acquired through chronic toxicity from topical medications,^19^ chemical or thermal injury,^20^ radiation,^21^ chronic ocular allergy,^22^ Stevens–Johnson syndrome,^23^ ocular cicatricial pemphigoid,^24^ graft versus host disease,^25^ contact lens wear,^26^^,^^27^ pterygium,^28^ and ocular surface tumors.^29^

LSCD has a very broad etiology, and statistical reports estimate that all forms of LSCD combined affect >5% of the world population. There are ∼125 million contact lens wearers worldwide, with 37 million in the United States.^30^ About 2.4% to 5% of those develop signs of LSCD,^31^^,^^32^ and ∼15% of the LSCD cases are attributed to contact lens use.^33^ This may be an underestimation, as mild cases are often asymptomatic and there are no clinical methods for early diagnosis of LSCD.^31^^,^^32^

Pterygium is a wing-shaped fibrovascular tissue proliferation from the conjunctiva onto the cornea, resulting from progressive LSCD caused by ultraviolet damage to the LSCs.^34^ As a pterygium invades the cornea, it causes loss of visual acuity, chronic irritation, recurrent inflammation, double vision, and impaired ocular motility.^35^^,^^36^ Pterygium occurs worldwide, and its incidence and prevalence vary by geographic location. For example, the prevalence of pterygium in the Black population in Barbados is 23.4%, but the prevalence of pterygium in Caucasians residing in urban, temperate climates (such as Canada) is ∼1.2%.^37^

Approximately 2.5 million people in the United States suffer an eye injury every year. Of the injuries leading to an emergency room visit, chemical burns account for up to 18% and thermal burns for ∼16%. Many of those patients develop LSCD, although the exact statistics remain unknown.^38^ Statistical data from the World Health Organization show that >400 million people worldwide suffer from visual impairment, ∼40 million are blind, and 25% of the cases are caused by corneal diseases or injury.^39^^,^^40^ Corneal disease-related blindness (including LSCD) has a significant negative impact on the quality of life of patients and results in a heavy social and economic burden. Corneal transplantation surgery allows for restoration of vision in patients with corneal-related blindness. However, the success of the transplantation is dependent on the presence of healthy LSCs in the limbus to allow for regeneration of the epithelium in the corneal graft. The limited availability of donor corneas, the high probability of immune rejection of graft corneas, and the lifelong administration of immunosuppressive drugs have limited the application of corneal transplantation, but novel corneal regenerative therapies based on LSC transplantation have become a topic of significant importance and research.^41^^,^^42^

Currently, the clinical diagnosis of LSCD relies on slit-lamp examination^3^ followed by impression cytology, which is an invasive method that requires skilled technicians. Although slit-lamp technology allows for non-contact imaging of the limbus over a very wide field of view (FOV), only the limbal surface can be viewed, and the spatial resolution is insufficient to resolve any cellular structure and other small morphological details. Cellular-level resolution images of the human limbus acquired in vivo with commercial confocal microscopes (IVCM) show en face views of the POVs and the cellular structure of the limbal epithelial crypts.^43^^–^^45^ Although the quality of these images is impressive, IVCM imaging has some limitations: (1) the imaging requires physical contact with the tissue surface, which can be painful or uncomfortable for the patient and increase the risk of potential infection or damage to the delicate limbal tissue; (2) axial scanning is too slow for the generation of volumetric images free of motion artifacts resulting from involuntary eye motion; and (3) the FOV of the en face images is limited to ∼400 µm × 400 µm.

Optical coherence tomography (OCT) allows for contactless, rapid, high-resolution, volumetric imaging of ocular tissue. The first application of OCT for in vivo imaging of the healthy human limbus was reported in 2011.^46^ The OCT images from that study showed the termination of Bowman's membrane, peripheral corneal nerves, limbal microvasculature, POVs, limbal crypts, and the Schlemm's canal. However, fine morphological details such as the shape of the POVs and the cellular structure of the limbal crypts were not resolved due to the limited axial and lateral resolution (3 µm and 18 µm, respectively). The same year, Li et al.^47^ published in vivo images of the heathy human limbal vasculature that were acquired using a 1300-nm OCT system and optical microangiography imaging protocol. In 2012, an ex vivo comparative study conducted on healthy limbal tissue with clinical confocal microscopy and a research-grade OCT system generated surface maps of the POVs from the OCT images.^48^ However, the cellular structure of the limbal tissue was not resolved due to the low OCT resolution (3.5 µm axial and ∼20 µm lateral). In 2015, a full-field OCT system with ∼1-µm isotropic resolution was used to image the limbal cellular structure, identify LSCs with the aid of fluorescence markers, and determine the size of the limbal crypts ex vivo in animal and human limbal tissue.^49^ During the period from 2017 to 2021, a number of research groups^50^^–^^54^ utilized commercial spectral-domain OCT (SD-OCT) systems (e.g., RTVue XR Avanti, RTVue RT-100-2) to image the healthy and pathological human limbus, including cases of LSCD. OCT images in these reports showed gross anatomy of the human limbus but failed to visualize fine morphological details, including the cellular structure of the limbal crypts due to the limited spatial resolution (>5 µm axial and >15 µm lateral) and low image acquisition rate (<50 kHz) of the commercial OCT devices. In 2017, our research group developed a high-resolution SD-OCT system (0.95 µm axial and ∼2 µm lateral), and reported the first, to our knowledge, in vivo, non-contact OCT images of the healthy limbus that showed the cellular structure of the limbal crypts, detailed shape of the POVs, and microvasculature of the underlying fibrous tissue.^55^ However, due to the slow image acquisition rate (34 kHz), volumetric images of the limbus acquired with this OCT system were compromised by significant eye motion artifacts. In 2022, our research group developed line-scan SD-OCT (LS-SD-OCT) technology and demonstrated its ability to generate in vivo volumetric images of the healthy human cornea and limbus that showed the cellular structure of these tissues in three dimensions.^56^ Although the LS-SD-OCT technology offers the advantages of rapid image acquisition, efficient suppression of eye motion artifacts, and clear images of the cellular structure of the limbal crypts, currently the clinical adoption of this technology is impeded by the large footprint of the system; by the complexity of its free-space design, which requires realignment by engineering personnel before each imaging session; and by the very high cost compared to point-scanning, fiber based SD-OCT technology. In this study, we utilized a compact, clinically viable, point-scanning SD-OCT technology with an improved image acquisition rate (250 kHz), developed by our research group, to acquire volumetric images of the healthy and pathological human limbus in vivo with ∼1.5-µm isotropic spatial resolution.

## Methods

### System Setup

 We recently developed a compact, fiberoptic, clinically viable, high-speed, ultra–high-resolution SD-OCT system designed for in vivo, contactless ophthalmic imaging, and we have demonstrated its ability to visualize the cellular structure of the human cornea.^57^ Briefly, the system is designed for operation in the 800-nm spectral region. It is powered by a femtosecond laser (INTEGRAL; Femtolasers, Wien, Austria) with an emission spectrum centered at 787 nm and a 3-dB spectral bandwidth of 138 nm. It offers ∼1.5-µm isotropic resolution in biological tissue assuming an average refractive index of 1.38. The system utilizes a customized, high-resolution spectrometer (Cobra; Wasatch Photonics, Morrisville, NC) connected to a line-scan camera (Teledyne e2v OctoPlus; Teledyne Technologies, Thousand Oaks, CA) with a tall pixel design, 2048-pixel array, and a maximum readout rate of 250 kHz. The signal-to-noise ratio (SNR) of the system was 98 dB measured at 100 µm away from the zero-delay line, with ∼10-dB roll-off over a 1.4-mm scanning range for ∼800-µW optical power of the imaging beam incident on the limbal surface. The imaging probe of the system is equipped with a microscope objective (Mitutoyo NIR infinity corrected series; Mitutoyo, Kanagawa, Japan). The choice of magnification power of the microscope objective determines the transverse resolution and the FOV of the OCT images. In this study, we utilized two such objectives: (1) 5×, nominal numerical aperture (NA) = 0.14, effective NA ∼ 0.07 (the entrance aperture was underfilled by ∼50%), resulting in transverse resolution of ∼4 µm and FOV of ∼3 mm × 3 mm; and (2) 10×, nominal NA = 0.26, effective NA ∼ 0.14 (the entrance aperture was underfilled by ∼40%), resulting in transverse resolution of ∼2 µm and FOV of ∼1.5 mm × 1.5 mm.

### Imaging Procedure

This study was approved by the Office of Research Ethics at the University of Waterloo and was carried out in compliance with the tenets of the Declaration of Helsinki. Subjects with LSCD of various etiologies (*n* = 4) and healthy controls (*n* = 6), ages 24 to 74 years, were recruited for this study. Both male and female subjects were included in this study; however, gender analysis of the data was not carried out because the main goal of this pilot study was to demonstrate the ability of the SD-OCT technology to visualize morphological features in the healthy and pathological limbus with cellular-level resolution. All subjects passed a slit-lamp biomicroscopy screening and provided written consent for participation in the study. A fixation target was used to align the eye so that the surface of the limbus was close to perpendicular to the imaging beam. Note that making the surface exactly perpendicular to the imaging beam will generate very strong back-reflections. Volumetric OCT images (1024 × 1024 × 1024 pixels) were acquired from the superior, inferior, and nasal limbus in both eyes, using two microscope objectives with 5× and 10× objectives to explore different combinations of FOV and transverse resolution.

### Image Processing

Images were generated from the raw OCT data and numerically dispersion compensated up to the fifth order with a custom MATLAB algorithm (MathWorks, Natick, MA). Volumetric and en face images were generated from the three-dimensional (3D) datasets using Amira Software (Thermo Fisher Scientific, Waltham, MA). Automatic alignment of B-scans (standard function in Amira) was used to compensate for involuntary eye motion before generation of the volumetric images.

### Participants’ Histories

The Table summarizes clinically relevant data for all participants in the imaging study. More detailed clinical histories are presented in the following paragraphs. Slit-lamp examination showed that control subjects H1 and H2 had healthy, normal corneas and limbus. Subject LSCD1 showed significantly increased pigmentation of the POVs (large number of melanocytes located along the inner walls of the limbal crypts). Subject LSCD1 was a 53-year-old Caucasian male diagnosed with pterygium in the left eye. Subject LSCD2 was a 74-year-old Hispanic male patient with LSCD in the left eye after excisional biopsy and adjuvant cryotherapy for a pigmented conjunctival tumor. The tumor was confirmed to be primary acquired melanosis with atypia. After excisional biopsy, the patient was treated with topical mitomycin C eye drops and subsequently developed a whorl-like keratopathy. A diagnostic biopsy was performed of this tissue which confirmed LSCD. After the OCT imaging session, the patient underwent surgical treatment with a selective limbal epithelial transplantation.

**Table.** Summary of the Clinically Relevant Data for All Study Participants

Subject Code	Sex	Age (Y)	Ethnicity	Clinical Diagnosis Based on Slit-Lamp Examination	Figure
H1	F	26	Middle Eastern	Healthy normal cornea and limbus	1B, 1D, 2, 3
H2	M	27	Asian	Healthy normal cornea and limbus	1C, 1E
LSCD1	M	53	Caucasian	Pterygium	4
LSCD2	M	74	Hispanic	LSCD after excisional biopsy and adjuvant cryotherapy in the right eye for a pigmented conjunctival tumor	5
LSCD3	M	24	Caucasian	LSCD secondary to atopic keratoconjunctivitis expressed in both eyes to different degrees	6
LSCD4	F	58	Asian	LSCD secondary to contact lens overwear (both eyes)	7

Subject LSCD3 was a 24-year-old male patient with LSCD secondary to atopic keratoconjunctivitis. He presented with 270° of LSCD, corneal neovascularization, and epithelial scarring on his right eye. His left eye was less involved, with only the superior 90° of the limbus affected. The patient improved with topical treatment using dexamethasone 0.1% (Maxidex; Alcon Canada, Mississauga, ON, Canada) and cyclosporine 0.05% (Restasis; Allergan, Markham, ON, Canada) eye drops. Subject LSCD4 was a 58-year-old female patient with LSCD secondary to contact lens overwear. The patient had been wearing contact lenses for over 40 years. She presented with 225° of LSCD in the superior limbus bilaterally. She improved with discontinuance of her contact lenses, topical treatment using fluorometholone 0.1% eye drops (FML; Allergan), and therapeutic superficial keratectomies.

## Results

**Figure 1.**
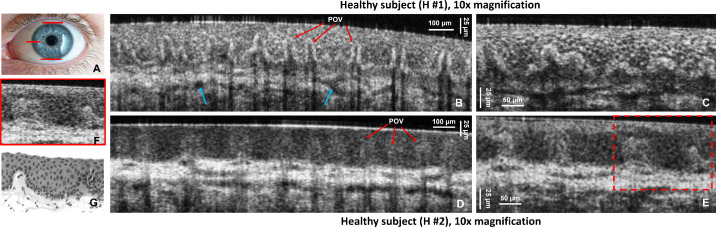
(**A**) Photograph of a human eye with *red lines* indicating the locations in the superior, inferior, and nasal limbus imaged with the OCT system. (**B**–**E**) Cross-sectional OCT images of the healthy human inferior limbus acquired from two healthy subjects (H1 and H2), showing the POVs (*red arrows*) and scleral vasculature (*blue arrows*). (**F**) Magnified view of the region of interest in panel E indicated by the *red dashed line*. (**G**) Typical histological image of a healthy human limbus showing POVs.

Figure 1A shows the locations in the inferior, superior, and nasal limbus from which cross-sectional and volumetric images were acquired (red lines). Figures 1B and 1D show cross-sectional OCT images of the heathy inferior limbus acquired from two of the healthy subjects: H1 and H2, respectively, over a length of ∼1.5 mm with the 10× microscope objective, resulting in ∼25% overlap between consecutive A-scans. Although cross-sectional views of the POVs are clearly visible in both images (red arrows), subject H1 had an unusually large number of pigmented cells (melanocytes) located along the inner boundaries of the limbal crypts which caused the POVs to appear to be of very high reflectivity. In contrast, the images acquired from subject H2 showed very sparse distribution of melanocytes, and the boundaries of the limbal crypts appeared to be of much lower reflectivity. OCT images acquired from the remaining four healthy participants in the study showed very similar appearance to the images acquired from subject H2 with one minor difference—the concentration and spatial distribution of melanocytes. All of the OCT images also revealed microvasculature located at the base of the POVs (blue arrows). Cross-sectional OCT images acquired from the same subjects and approximately the same locations in the limbus over a length of ∼0.5 mm with the 10× microscope objective, resulting in ∼75% overlap between consecutive A-scans, revealed the cellular structure of the limbal crypts (Figures 1C, 1E). In order to identify properly morphological features observed in the OCT images, we compared them with histology of a healthy, normal limbus. A magnified view of the region of interest in Figure 1E marked with the dashed red line is shown in Figure 1F, and a typical histological image of the POVs in the healthy human limbs is displayed in Figure 1G.

**Figure 2.**
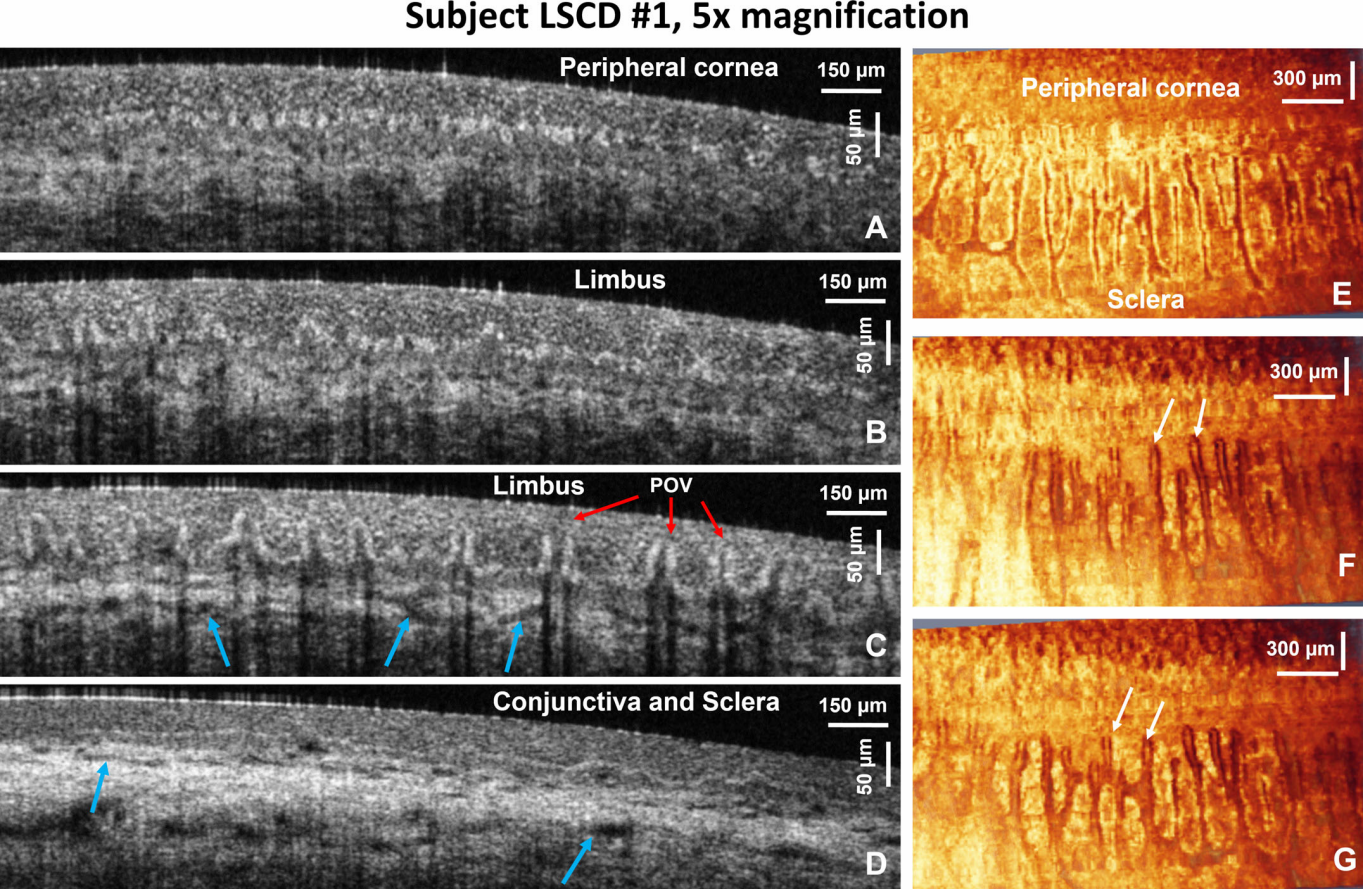
OCT images acquired from subject H1. (**A**–**D**) Large FOV (3 mm) cross-sectional OCT images of the healthy human inferior limbus showing the POVs (*red arrows*) and scleral vasculature (*blue arrows*). (**E**–**G**) En face images of the limbus generated at depths of approximately 15 µm, 30 µm, and 45 µm below the tissue surface revealing structures under the folds of the POV. The *white arrow* indicates capillaries under the folds of the POVs.

Figure 2 shows selected cross-sectional and en face images of the healthy human limbus that were acquired from subject H1 over a large FOV (3 mm × 3 mm) with the 5× microscope objective. Figures 2A to 2D show representative B-scans from a 3D image set that show how the limbal morphology varies from the peripheral cornea to the conjunctiva and sclera. Figures 2E to 2G show en face OCT images of the limbus that were generated from the volumetric dataset for depths of approximately 15 µm, 30 µm, and 45 µm below the tissue surface. These images present en face cross-sections of the POVs and reveal hyperreflective, string-like structures under the folds of the POVs (Figs. 2F, 2G) that, according to limbal anatomy, most likely correspond to capillaries running radially under the folds of the POVs.

**Figure 3.**
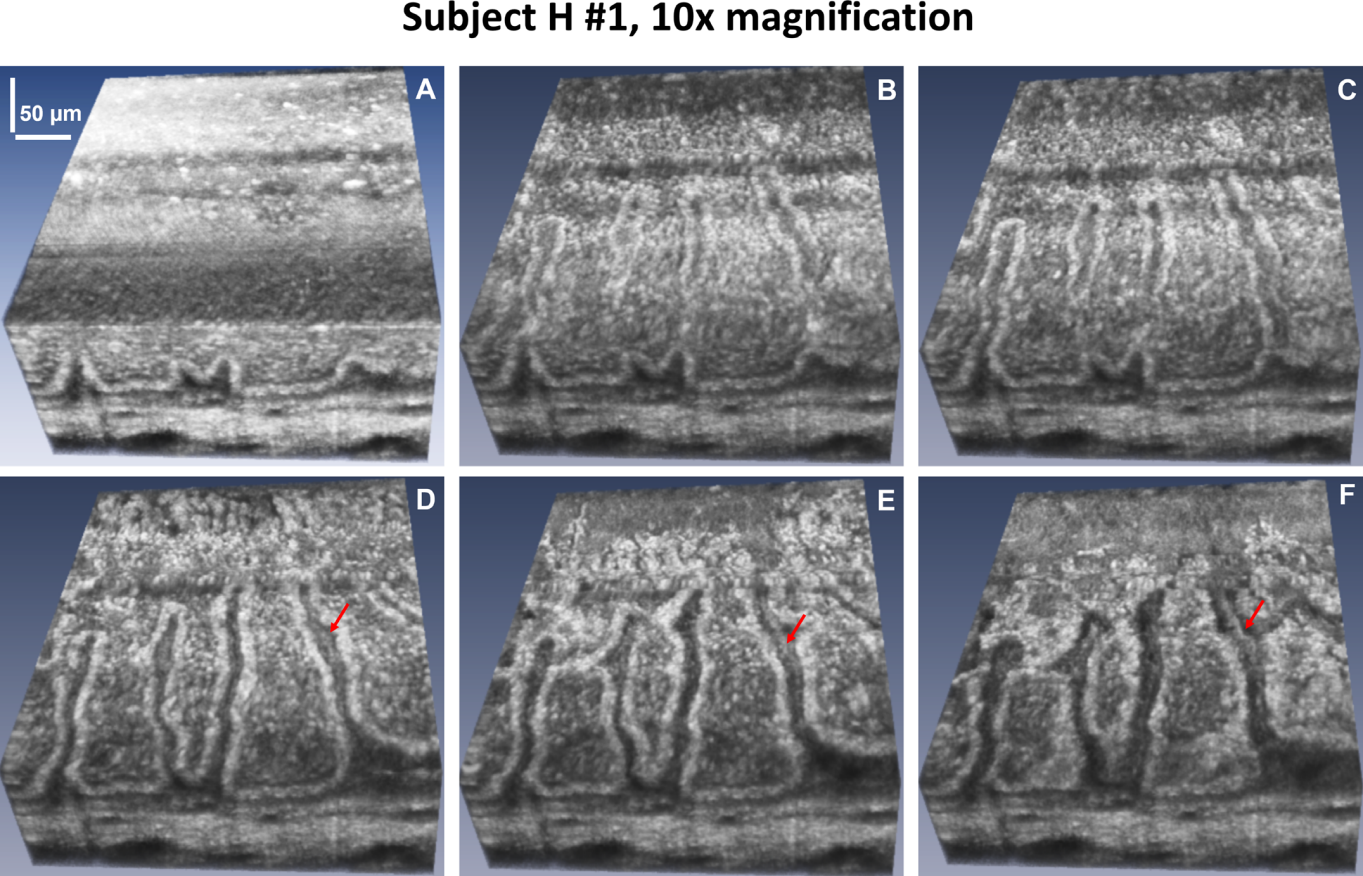
(**A**) High-resolution volumetric OCT images of the healthy inferior limbus acquired from subject H1. (**B**–**F**) En face images generated from the 3D image stack for different depths that reveal the cellular structure of the limbal crypts and limbal capillaries running radially under the folds of the POVs (*red arrows*).

Figure 3A shows a 3D OCT dataset that was acquired from subject H1 over a FOV of approximately 400 µm × 400 µm of the healthy inferior human limbus with the 10× microscope objective (∼80% overlap between consecutive A-scans). The series of en face images (Figs. 3B–3F) revealed the volumetric structure of the limbal crypts with cellular resolution, as well as the 3D structure of the POVs. The red arrows indicate the hyperreflective, string-like structures located under the folds of the POVs.

**Figure 4.**
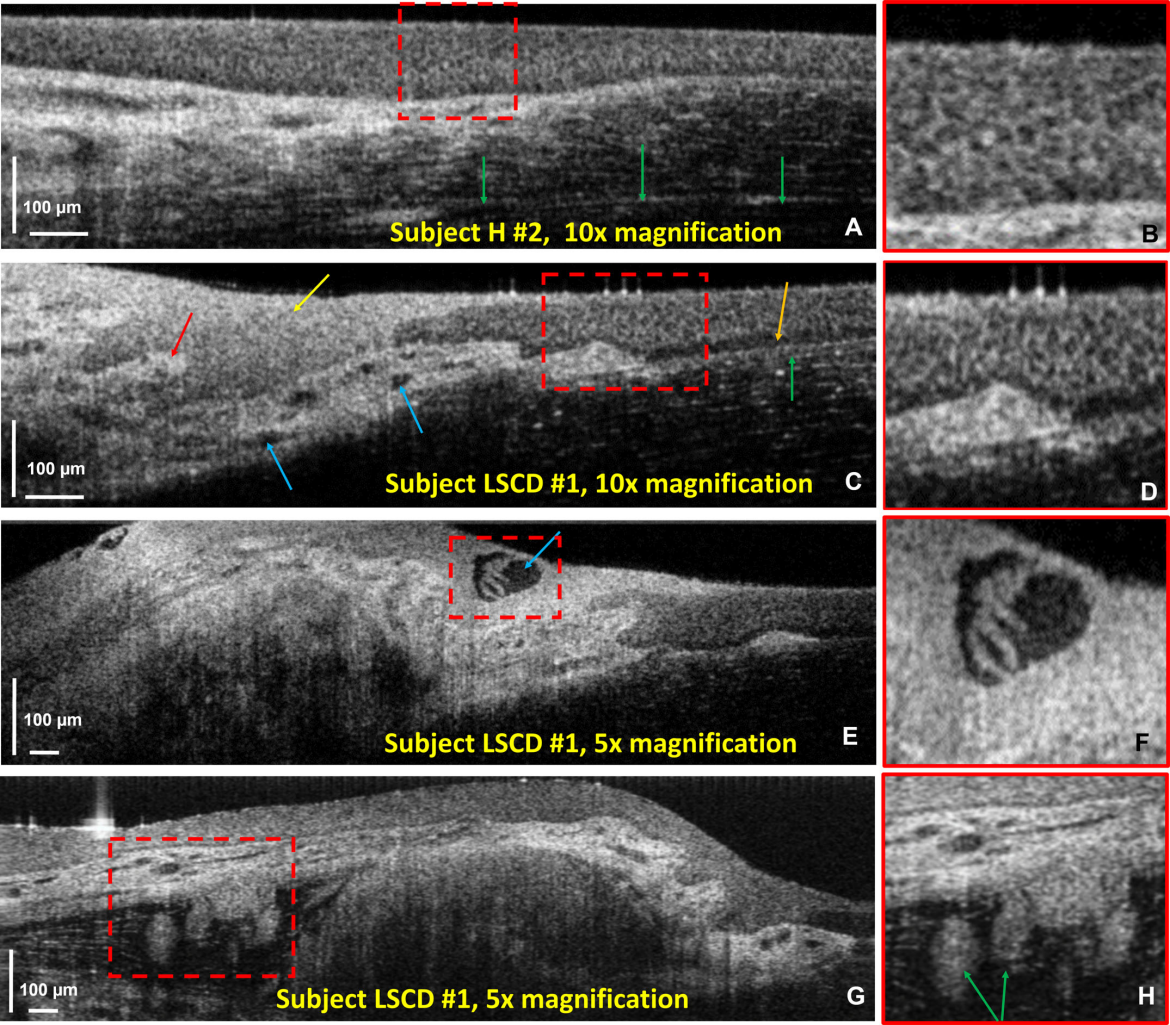
OCT B-scans of the nasal limbus acquired from subjects H2 and LSCD1. (**A**, **B**) Healthy limbus showing the cellular structure of the corneal epithelium and a large peripheral corneal nerve (*green arrows*). (**C**–**H**) Images of pterygium characterized with overgrowth of highly scattering conjunctival tissue, neovascularization (**C**, *blue arrows*), cysts (**F**), and glands (**H**, *green arrows*).

Figure 4 shows a collection of cross-sectional OCT images acquired from the nasal region of the limbus of a healthy subject (H2) (Figs. 4A, 4B) and a patient with pterygium (LSCD1) (Figs. 4C–4H). Figures 4A and 4B show the cellular structure of the epithelial layer in the peripheral cornea and the limbus. A cross-section of a large peripheral corneal nerve is also visible in Figure 4A (green arrows). Figure 4C shows an OCT B-scan that was acquired from a similar location in the nasal limbus from a patient with pterygium. The cellular structure of the corneal epithelium, basal cell layer (orange arrow), and Bowman's membrane (green arrow) are clearly visible in the healthy part of the peripheral cornea. The conjunctival tissue growing over the limbal region (yellow arrow) appears more scattered, and its cellular structure is less clearly visible. The pterygium is highly vascularized, and cross-sections of blood vessels are indicated in the image with the blue arrows. The vasculature appears to be surrounded by tissue (red arrow) that is much more scattered than the normal conjunctiva. Figure 4D shows hyperreflective growth that appears to be located underneath the basal cell layer of the normal corneal epithelium. Figures 4E and 4F show a large cyst filled with clear fluid and tissue growth visible inside. Figures 4G and 4H show glandular-like morphological features that are located underneath the highly vascularized layer (Fig. 4H, green arrows).

**Figure 5.**
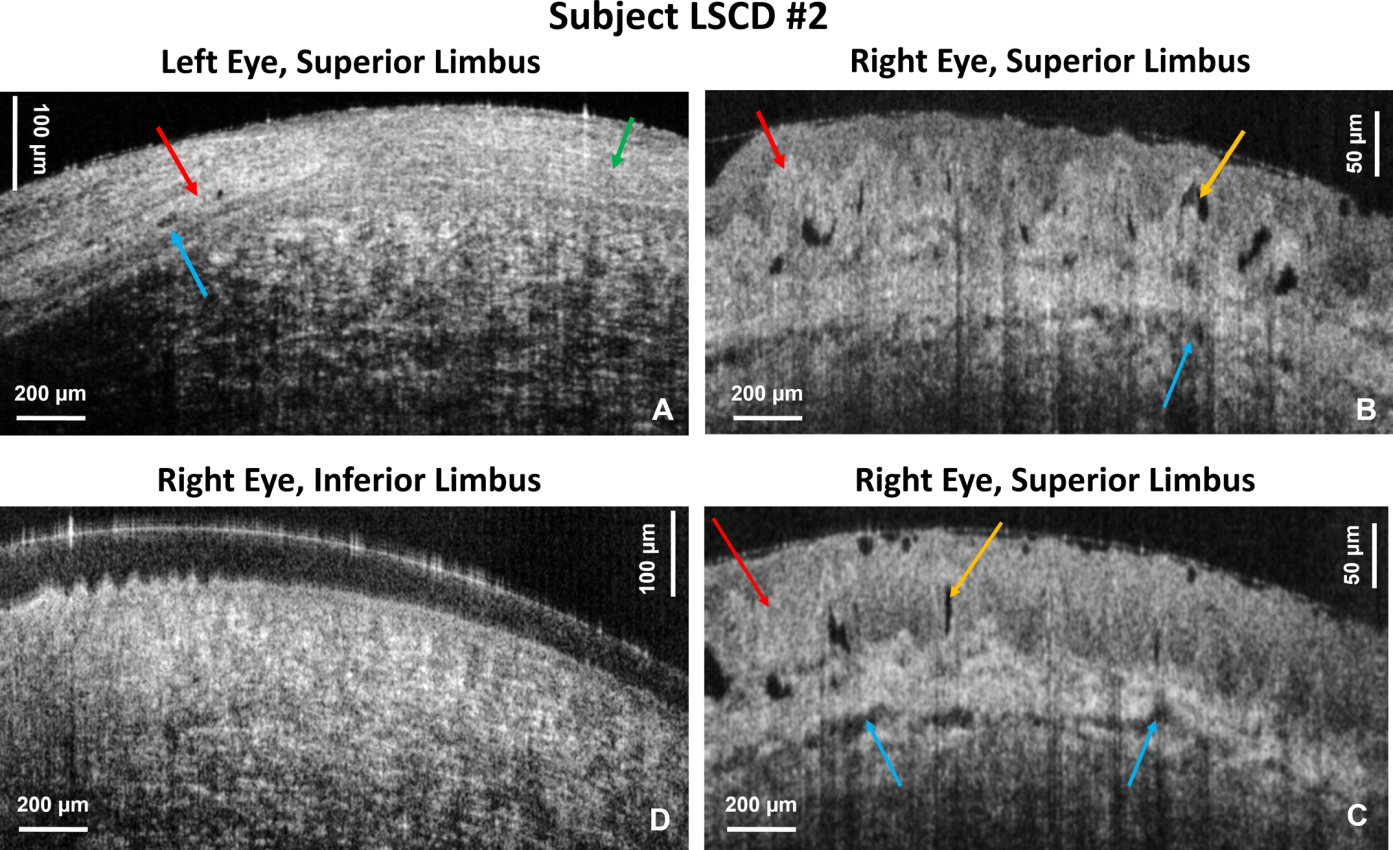
OCT cross-sectional limbal images acquired from subject LSCD2 with the 5× objective. (**A**) Image of the superior limbus in the left eye after excisional biopsy and adjuvant cryotherapy for a pigmented conjunctival tumor. *Red arrows* indicate highly scattered tissue surrounding the blood vessels (*blue arrow*). (**B**, **C**) OCT images of the superior limbus of the right eye. The *red arrow* indicates highly scattered scar tissue, the *orange arrow* indicates a cyst in the POVs, and the blue arrow indicates microvasculature in the underlying tissue. (**D**) This image shows normal appearance of the superior limbus.

Figure 5 shows cross-sectional images acquired from subject LSCD2 with the 5× objective. Figure 5A shows an OCT B-scan of the left superior limbus that was acquired after excision of the conjunctival tumor and adjuvant cryotherapy treatment and before the patient underwent selective limbal epithelial transplantation. The POVs and limbal crypts are missing and have been replaced with scar tissue from the surgery (green arrow). The tissue bordering the surgical scar shows neovascularization with blood vessels (blue arrows) surrounded by hyperreflective tissue (red arrow). Under slit-lamp observation, the right eye superior limbus appeared clinically normal. Cross-sectional OCT images of the same region (Figs. 5B, 5C) show well-defined POVs and limbal crypts. However, the POVs appear distorted in shape, surrounded by a thick layer of highly reflective tissue (red arrow), and they contain cysts filled with clear fluid (orange arrow). Figure 5D shows the normal appearance of the transition from the peripheral cornea (right side) to the proximal limbus (left side where tips of the POVs are visible) in the inferior limbus of the patient's right eye.

**Figure 6.**
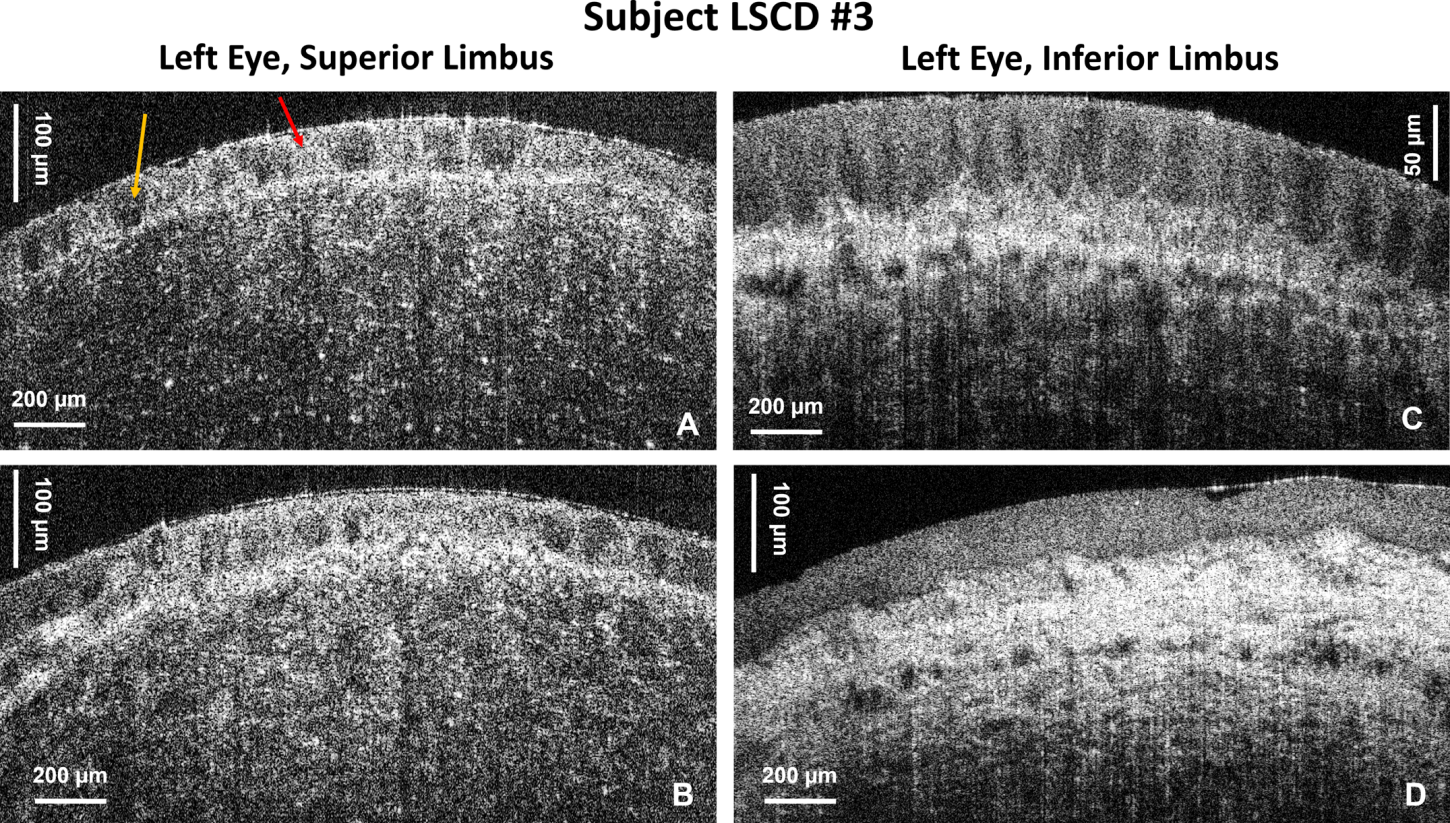
OCT cross-sectional limbal images acquired from subject LSCD3 secondary to atopic keratoconjunctivitis with the 5× objective. (**A**, **B**) Images from the superior limbus of the left eye. *Red* and *orange arrows* indicate highly scattered and low-scatter regions in the scarred epithelium, respectively. (**C**, **D**) Images of the inferior limbus of the left eye show normal appearance of the limbal POVs and crypts and the transition to the conjunctiva and sclera.

**Figure 7.**
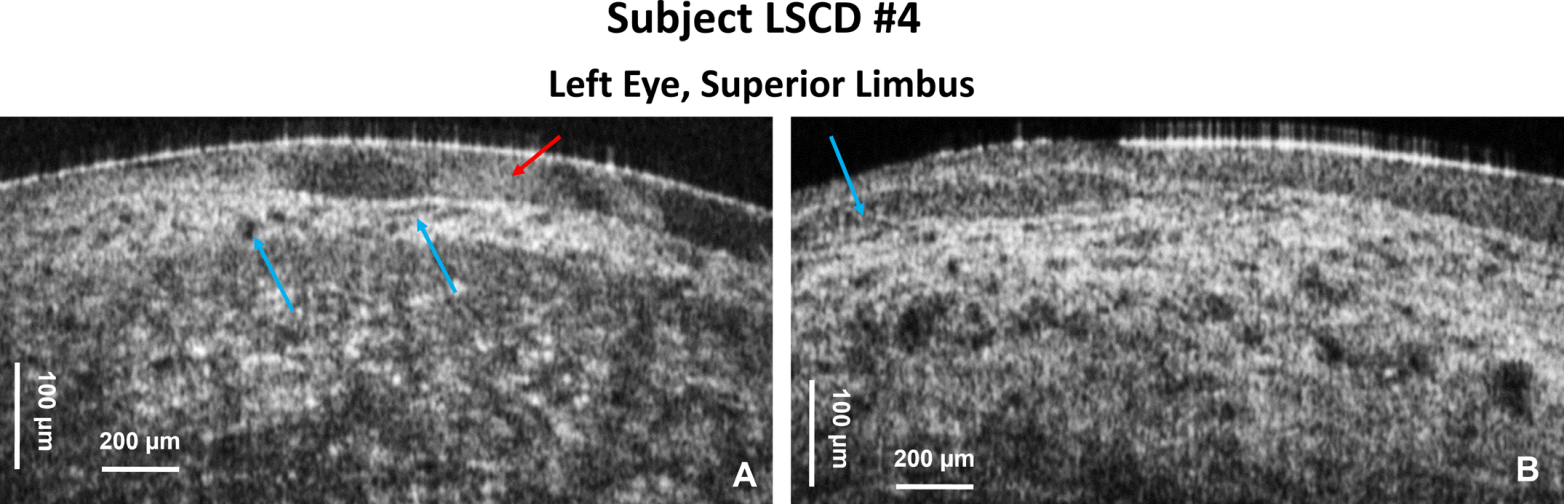
(**A**, **B**) OCT cross-sectional limbal images acquired from subject LSCD4 secondary to contact lens overwear with the 5× objective. The *red* *arrow* indicates highly scattered regions in the scarred epithelium, and the *blue* *arrows* indicate neovascularization.

Figure 6 shows limbal OCT images acquired from subject LSCD3 with the 5× objective. Figures 6A and 6B show images from two locations in the superior limbus of the left eye that exhibit epithelial scarring (red arrows) and thinning. An image of the inferior limbus of the same eye (Fig. 6C) shows almost normal appearance of the POVs and limbal crypts. An image of the transition from the distal end of the limbus to the conjunctiva and sclera (Fig. 6D) acquired from the left eye also shows normal appearance of the tissue. Figure 7 shows an OCT image acquired from the inferior limbus of the left eye of subject LSCD4 with the 5× objective. The OCT images show a lack of POVs and limbal crypts in the inferior limbus, scarring of the epithelium (red arrow), and neovascularization (blue arrows).

## Discussion

Although OCT has been used to image in vivo the morphology of the human limbus in both health and disease for the past 13 years,^46^^–^^56^ the limited spatial resolution and the slow image acquisition rate relative to the speed of ocular saccades have prevented volumetric visualization of limbal cellular structure in most of those studies. To the best of our knowledge, so far our research group has been the only one to image in vivo individual cells in the limbal crypts, as well as the 3D structure of POVs in a contactless way and with image quality comparable to that of IVCM with both point-scanning^55^ and line-scanning SD-OCT.^56^ Because the average size of stem cells and progenitor cells in the limbal crypts is ∼10 µm or larger, whereas corneal cells can range from approximately 15 µm to 35 µm in diameter depending on the corneal layer (basal cells, wing cells, or superficial cells),^60^ OCT technology with spatial resolution equal to or higher than 2 µm is sufficient to visualize both individual cells in the human cornea and limbus and reflections from their nuclei. However, special resolution alone is not sufficient for resolving individual cells in OCT images. If the OCT image acquisition rate is slow compared to fast involuntary eye motion, ocular saccades will introduce blur in the OCT images that can compromise the ability to resolve individual cells in the OCT images. This is the case with the point-scanning SD-OCT technology developed by our research group^55^^,^^61^^,^^62^ back in 2016, which offered spatial resolution of <1 µm and a slow scanning speed (34,000 A-scans per second, equivalent to ∼33 frames per second [fps]), which introduced too many eye-motion–related image artifacts and blur and severely restricted the FOV. This prompted the development of a faster, high-resolution point-scanning OCT technology to be used for clinical evaluation of LSCD.

Here, we have presented an upgraded version of a point-scanning SD-OCT technology that strikes a balance by combining high spatial resolution (∼1.5 µm) and sufficiently high image acquisition rate (250,000 A-scans per second, equivalent to ∼244 fps) to suppress most of the eye-motion–related image artifacts. Point-scanning SD-OCT offers the advantages of compact size, simple alignment, easy operation by non-engineering personnel, high spatial resolution, and high SNR. However, even at the significantly improved data acquisition rate (250,000 A-scans per second), ocular saccades can still distort the volumetric OCT images and compromise the sharp appearance of the cellular membranes of the cells populating the limbal crypts. In cases when imaging is conducted with a high-magnification objective corresponding to a short depth of focus, volumetric images acquired with the 250-kHz point-scanning SD-OCT system can still be occasionally compromised by axial microsaccades, which can shift the tissue out of focus and change the OCT image contrast. An example of such artifacts can be observed in Figures 3A to 3C. The darker horizonal stripes are due to axial shift of the limbus relative to the focal plane of the microscope objective, which caused a reduction in the image contrast.

In cases when the imaged subject has poor fixation ability, larger saccades can shift and twist consecutive OCT B-scans and prevent software products such as ImageJ and AMIRA from generating volumetric and en face images from the volumetric datasets. In our case, all four LSCD subjects had poor vision and great difficulty with fixation, which resulted in the OCT volumetric dataset being compromised by strong motion artifacts. Therefore, here we can only show individual cross-sectional OCT images. However, a simple run through the cross-sectional images from a volumetric dataset can still offer a clinician plenty of information on how the LSCD limbal morphology varies over the imaged region.

LS-SD-OCT technology operating at an image acquisition speed of ∼2400 fps was able to generate volumetric images of the healthy human limbus of impressive quality and nearly free of eye motion artifacts.^51^ However, the translation of LS-SD-OCT to wide clinical use is currently impeded by the large footprint, high cost, and the requirement of engineering personnel to realign the system before each day of imaging sessions. Volumetric images acquired with this system from both healthy (Figs. 1^2^–3) and pathological (Figs. 4^5^,^6^–7) human limbus demonstrate that this technology is capable of mapping the POVs over a fairly large FOV (∼3 mm × 3 mm), while still being able to resolve individual cells in the limbal crypts. In the case of pterygium (Fig. 4), the system was able to visualize microvasculature imbedded in tissue that has higher scattering profile compared to the conjunctiva, as well as cysts and glands. Although OCT has been used in the past to image pterygium,^58^^,^^59^ the low spatial resolution combined with slow imaging rate has prevented visualization of fine morphological details.

In the case of conjunctival tumor removal (Fig. 5A), the fast SD-OCT system was able to image the string-like structure of the scar region, as well as the neovascularization at the scar boundary. The patient's right eye appeared clinically normal on examination with slit-lamp bio-microscopy; however, on the cross-sectional OCT images, the POVs appear distorted with a thick layer of reflective tissue. Note that slit-lamp microscopy generates only en face images of the limbus with very low spatial resolution. The OCT generates cross-sectional images that reveal the limbus morphology in depth with very high spatial resolution. Therefore, we conclude that, although the slit-lamp examination showed normal appearance of the limbus, the high-resolution cross-sectional OCT images revealed altered morphology compared to images acquired from healthy, normal subjects. In the cases of atopic keratoconjunctivitis (Fig. 6) and LSCD caused by extensive wear of contact lenses (Fig. 7), complete loss of the POVs was observed in the affected eyes and instead the limbal region shows scar-like regions of high optical reflectivity alternating with low reflective regions.

The LSCD patients imaged for our study did not undergo IVCM examination as part of their clinical diagnosis or as participants in this study because IVCM technology was not available in the clinic. Therefore, we cannot provide a direct comparison between OCT and IVCM images for the LSCD participants in our study. However, we did compare the OCT images with histology for the healthy, normal limbus (Fig. 1; see also our recent publication regarding the LS-SD-OCT system^56^), which allowed us to identify the appearance of the POVs and limbal crypts in the OCT images of the healthy, normal limbus.

Direct comparison of IVCM and low-resolution OCT images of the healthy human limbus, identifying the same morphological features in both types of images, was also reported by another research group.^50^ The major advantage of the high-resolution, fast point-scanning SD-OCT technology developed by our research group and presented here is the ability to generate volumetric images of the limbus with cellular resolution over a wider FOV in a contactless method. In contrast, IVCM requires physical contact with the tissue and can only generate en face images because volumetric image acquisition is compromised by eye-motion artifacts. Furthermore, due to the high spatial resolution, the FOV of IVCM is typically restricted to <400 µm × 400 µm, whereas high-resolution OCT images with our OCT system can be acquired from a significantly larger FOV (>3 mm × 1 mm).

The use of point-scanning SD-OCT technology to image corneal limbal stem cells has the potential to clinically evaluate and quantify the health and viability of the tissue in a non-invasive manner. So far, in this pilot study, we demonstrated that fast, high-resolution, clinically viable point-scanning SD-OCT can visualize the POVs and cellular structure of the limbal crypts and limbal vasculature in healthy limbus, as well as scarring, cysts, and neovascularization in limbus affected by LSCD. Both healthy and diseased limbus can be characterized by different metrics associated with the OCT images: size, shape, and density of the POVs; size and shape of the limbal crypts; cell density of the cells populating the limbal crypts; optical density of the highly scattering tissue in conditions such as pterygium; changes to the vascular maps of the limbal vasculature; and presence, shape, and density of cysts, among others. When clinical studies have been conducted with high-speed, high-resolution point-scanning SD-OCT technology on a large number of LSCD subjects, novel image processing algorithms based on machine learning and artificial intelligence can be developed to extract and quantify relevant morphological features from the volumetric OCT datasets The possibility of integrating the OCT technology with a surgical microscope would provide live, real-time imaging, thus optimizing surgical approaches and precision for the treatment of LSCD and other ocular surface conditions that may lead to corneal blindness.

## Conclusions

In summary, we have demonstrated that point-scanning SD-OCT technology, which combines high spatial resolution, relatively high image acquisition rate, and a compact design at moderate cost, is able to image the human limbus in vivo, volumetrically, in a contactless way, while still allowing for visualization of the cellular structure of the limbal crypts. Furthermore, we have demonstrated the clinical potential of this technology for the assessment of patients with LSCD.
